# Key Developments in Ionic Liquid Crystals

**DOI:** 10.3390/ijms17050731

**Published:** 2016-05-16

**Authors:** Alexandra Alvarez Fernandez, Paul H. J. Kouwer

**Affiliations:** 1 Institute for Molecules and Materials, Radboud University, Heyendaalseweg 135, 6525 AJ Nijmegen, The Netherlands; a.alvarez@ntu.edu.sg; 2 School of Materials Science and Engineering, Nanyang Technological University, Blk N4.1 Nanyang Avenue, Singapore 639798, Singapore

**Keywords:** ionic liquid crystals, liquid crystals, ionic liquids, imidazolium, smectic

## Abstract

Ionic liquid crystals are materials that combine the classes of liquid crystals and ionic liquids. The first one is based on the multi-billion-dollar flat panel display industry, whilst the latter quickly developed in the past decades into a family of highly-tunable non-volatile solvents. The combination yields materials with a unique set of properties, but also with many challenges ahead. In this review, we provide an overview of the key concepts in ionic liquid crystals, particularly from a molecular perspective. What are the important molecular parameters that determine the phase behavior? How should they be introduced into the molecules? Finally, which other tools does one have to realize specific properties in the material?

## 1. Introduction

Most materials show a transition from a crystalline phase into an isotropic liquid phase. Some materials, however, melt into anisotropic fluids. The state of matter of these ordered liquids has been described as ‘in between a crystalline solid phase and a fluid isotropic phase’ and is commonly referred to as a liquid crystal (LC) phase or mesophase [1,2]. The materials that form these intermediate phases are referred to as liquid crystals or mesogens. The class of ionic liquid crystals (ILCs) merges the classes of LCs and ionic liquids (ILs). In this relatively new family of materials, the properties are a true combination of both components: anisotropy and fluidity of LCs and the designer solvent properties of ILs. This review covers recent developments in the field of ILCs, a field that has rapidly expanded in the past years. Rather than giving a full overview of all presented molecules and their liquid crystalline properties, we aim to capture the major developments. Before discussing ILCs in Paragraph 4, we will introduce some of the important concepts of liquid crystals in Paragraph 2 and provide some general remarks on ILCs in Paragraph 3.

This review follows two important reviews from Binnemans [3] and Laschat [4]. The former extensively summarizes all the work in on ILCs carried out until 2005. The second focuses on new materials developed between 2005 and 2011 and is an update of the work of Binnemans. Both excellent publications structure and classify the ILC field based on the chemical nature of the cationic group.

Over the past decade, the field of ILCs has rapidly developed and a review of all these new materials may easily become too broad. Instead, we focus on the key steps that have advanced the field with an additional emphasis on the steps that need to be taken in the future to advance this class of materials towards applications. In line with the aforementioned reviews, we structure our manuscript based on the (organic and sometime liquid-crystal-shaped) cation. The scope of this review is limited to thermotropic ionic liquid crystals and does not include closely related lyotropic and chromonic liquid crystals (that form mesophase in the presence of a solvent), although some of the compounds discussed in this work may also form such mesophases. For more information on these classes of LCs, we direct the reader to some excellent reviews [5,6,7,8,9]. In addition, the class of metallomesogens, which often also display an ionic character is not included in this work and is reviewed elsewhere [10].

## 2. Liquid Crystals: The Fourth State of Matter

The field of liquid crystals has an extensive history. Already in the 19th century, LCs were described and interpreted as ordered, but fluid materials [11,12]. After that though, it took many years to develop from an academic curiosity to the current multi-billion-dollar flat panel display industry.

**Figure 1:**
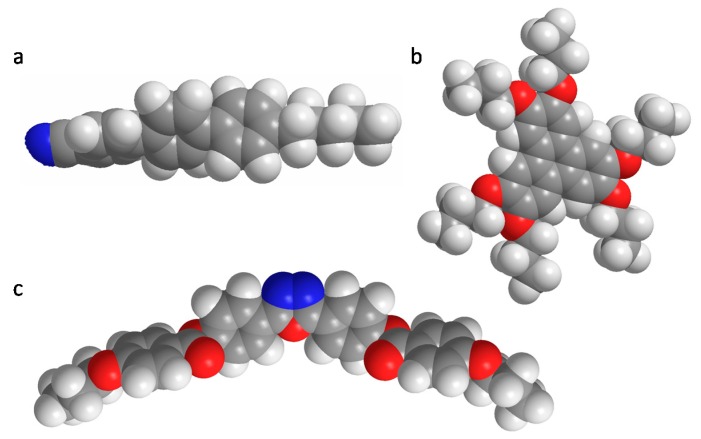
Spacing-filling models of archetypical mesogens: (**a**) rod-shaped terphenyl derivative; (**b**) disc-shaped triphenylene derivative; and (**c**) diphenyloxadiazole-based bent-core mesogen. Note that the aliphatic tails have been shortened for clarity. Atom color coding: black: carbon; white: hydrogen; green: nitrogen; and red: oxygen.

Researchers classify LCs in different ways. One common way is a classification by their molecular shape. Many liquid crystals are built-up from a rigid, often aromatic core that is substituted by flexible aliphatic tails. The shape of the molecule often determines the type of mesophase that is observed. The majority of known mesogens are rod-shaped or calamitic (Figure 1a) and these molecules are preferentially used in switching applications. Other classes are disc-shaped or discotic mesogens (Figure 1b) and bent-core or banana-shaped mesogens (Figure 1c). In all examples, the molecular anisotropy is clear and is one of the key driving forces for mesophase formation. Other interactions that play an important role are dipole-dipole interactions, π–π stacking and Van der Waals interactions.

**Figure 2:**
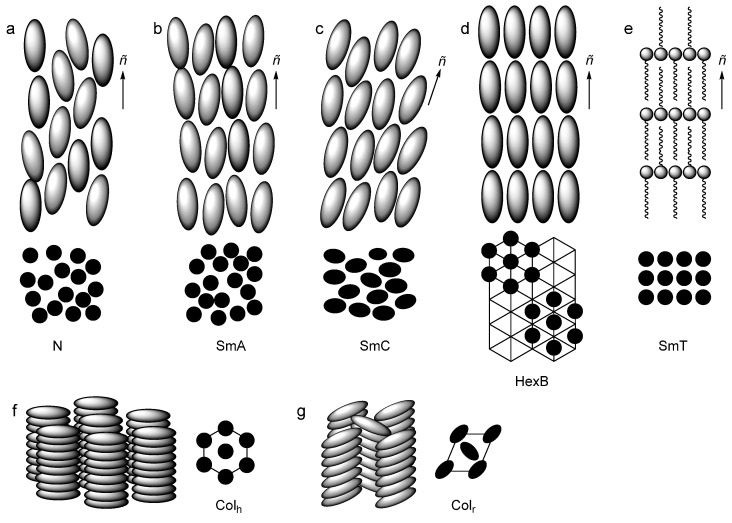
Schematic representation of a selection of frequently observed liquid crystal phases (**a**–**d**) for rod-shaped LCs; (**f**,**g**) for disc-shaped LCs and (**e**) selectively for ILCs. For all phases a side view (grey) of the phase and a top view (black) are given. The director *ñ* is indicated for every mesophase and is tilted with respect to the layer direction in the SmC phase. Phase abbreviations: N = nematic; SmA, SmC, SmT = smectic A, C and T, resp.; HexB = hexatic B; Col_h_, Col_r_ = columnar hexagonal and rectangular, respectively.

A second, fundamentally better classification is based on the type of mesophase that the molecules form, *i.e.*, the symmetry of the mesophase. For this, we need to realize that the molecular arrangement within a liquid crystal phase is described by two different levels of order. The orientational order describes the orientation of the molecules to their average orientation, the director, which is described by a unit vector *ñ*. Positional order describes the position of the molecules with respect to a (three dimensional) lattice. With different levels and dimensions of order, different mesophases can be formed. The main classes are the nematic (N) phase, in which the molecules only exhibit orientational order, and the smectic (Sm) and columnar (Col) phases that in addition to orientational order also show long-range positional in one or two dimensions, respectively. Here the director *ñ* is defined perpendicular to the layers or parallel to the columns. These main groups of mesophases, however, can be further subdivided into many phases with slight chiral effects, different in-layer organization and twists and multi-layer super-organizations into a wide range of mesophases [13]. Figure 2 shows some of the frequently found mesophases.

The nematic (N) phase (Figure 2a), frequently found for calamitic molecules is characterized by only orientational order along the director *ñ*, which is a unit vector of the average molecular orientation in a LC domain. The addition of a chiral dopant to the nematic phase, or the use of a chiral mesogen may lead to the formation of a chiral nematic or cholesteric (N*) phase, wherein the molecules again only possess orientational order, but additionally are arranged in a helical manner. The N and N* phases are common phases for display applications, since they benefit from the low viscosity, associated to the low order in the phase, which decreases the switching rates. In addition, disc-shaped and bent-core mesogens are known to form nematic phases, but often elevated temperatures are required.

Phases with orientational order and one-dimensional positional order along the director *ñ* are called smectic phases. There are different types of smectic (or layered) mesophases that differ in the in-layer molecular organization; their nomenclature is based on a historic assignment and is not related to the exact type of organization in and between the layers. The smectic A (SmA) phase (Figure 2b) shows such layered assembly wherein the mesogens are oriented with their long axis parallel to the director but lack in-plane positional order. As we will see later in this review, the SmA phase is predominantly found for ionic liquid crystals as Coulombic forces and strong micro-segregation dominate the intermolecular interactions. Consequently, also very few examples of nematic phases of ILCs have been reported [14,15,16]. One can further subdivide the SmA phases by closer investigating the ‘unit cell’ of the material. For instance, X-ray diffraction analysis showed that the molecules in some materials, including ILCs, actually form bilayers and thus formally show a bilayered SmA (SmA_2_) phase. Similarly, one can also distinguish interdigitation, layer undulations and other super-structures to further define the order in the layered phase.

The smectic C (SmC) phase (Figure 2c) differs from SmA phase as the mesogens in the layers show an average tilt with respect to the layer normal, but still lack in-plain positional order. The hexatic B (HexB) phase (Figure 2d) is characterized by in-layer a short-range positional order in addition to the layered order. There is a smectic phase that has been only found in ILCs [17], the smectic T (SmT) phase (Figure 2e). It is characterized by tetragonal layers separated by long alkyl chains and is formed by ILCs based on ammonium mesogens.

Discotic molecules tend to organize into columns. Depending on the subsequent two-dimensional arrangement of these columns, one can distinguish two major types of mesophases; hexagonal (Col_h_) and rectangular (Col_r_) columnar phases (Figure 2f,g). In a second index, one can indicate the order within the columns. Cubic (Cub) phases (not shown) have no orientational order, but do display three-dimensional positional order and are rarely observed for ILCs.

All different types of mesophases have a common factor: order and anisotropy from the molecular to the micron scale. This macroscopic anisotropy introduces anisotropy in many physical properties. In other words, bulk properties like the refractive index, magnetic susceptibility, dielectric permittivity, *etc.* show different values as a function of the orientation to the director *ñ* (either parallel or perpendicular to it) [18]. These unique properties form the basis of display applications [19] or, more broadly, for virtually any application that involves liquid crystals, such as actuators, sensing, micro-optics, templates and many other fields [20,21,22,23,24,25,26,27,28].

## 3. Ionic Liquid Crystals: Merging Liquid Crystals and Ionic Liquids

The default molecular design of a (calamitic or discotic) mesogen can be modified by the introduction of ionic groups leading to the formation of mesomorphic salts called ionic liquid crystals [29]. Their ionic character makes them closely related to another class of emerging materials, ionic liquids [30,31,32]. ILCs offer the features of ILs with the additional benefits of anisotropy [3,4], and truly are a merge of the two components. This may be best reflected by the different views on the field that researches have depending on their background: anisotropic ILs, or LCs with charged groups [33,34]. In either case, the development of ILCs opened up new avenues in both research fields [35,36,37,38].

In general, the molecular design of an ILC consists of an organic cationic core, most often based on a quaternary nitrogen atom, which is attached to a long hydrophobic chain and paired with an (inorganic) anion. The driving forces for the mesophase formation and stabilization of ILCs include (besides the aforementioned interactions) micro-phase-segregation of incompatible units (the charged and neutral parts of the molecule) as well as electrostatic interactions between cations and anions [3]. The first example of an ionic liquid crystalline material was given by Ujiie and coworkers [39]. Compared to analogous neutral mesogens, their ammonium-based ILCs showed both lower melting temperatures *T*_m_ (transition from the crystal to LC phase) and higher clearing temperatures *T*_c_ (transition from the LC to the isotropic phase), leading to an overall stabilization of the mesophase.

Starting from an unknown ionic mesogen, there is no conventional method to predict the type of mesophase the compound will form or the thermal range of the mesophase stability. This vacancy in the field can be attributed mainly to a lack of understanding of the many molecular factors that influence the stability of the (ionic) mesophase. There are, however, significant experimental efforts in the field that aim to develop such set of rules to predict the thermal range of ionic liquid crystals [40]. Important parameters in such studies are for instance the type of cationic core, the length and nature of a substituted aliphatic chain and the counter ion. In the case of the counter ion, which obviously plays a crucial role in ILCs, the standard approach was to pair the mesogenic cationic core with small spherical inorganic anions. Recently, anions with a strong organic character have shown outstanding properties for the thermal stability and the reduction of the phase transition temperatures [16,41].

As in conventional uncharged mesogens, it remains difficult to use computational methods in the prediction of phase behavior. In 2010, a theoretical approach was reported [42]. By employing density functional theory, the authors attempted to explain the influence of the anisotropic charge distribution on ionic mesophase stability. The work predicted that the stability range of nematic phases in ILCs is rather small, but, importantly, not negligible. The authors concluded that the micro-segregation effect of incompatible charged (cation or anion) and neutral moieties plus the aggregation of compatible hydrophobic chains leads to a layered structure of alternating charged and hydrophobic groups. A stable nematic phase can then be induced by increasing the strength of the electrostatic interactions, for instance by placing a single charge in the center of the mesogen or two charges on a certain distance.

Molecular Dynamics (MD) simulations are well-known tools to increase the understanding of condensed matter. Both LCs and ILs have been intensely studied in the development of force fields for classical MD simulations [43,44]. In the case of ILCs, the long range order and electrostatic interactions significantly increase the viscosity compared to traditional ILs and the development of an entire phase diagram requires extensive simulations to properly equilibrate the system [45]. In 2012, Saielli reported the thermal behavior of a model system based on a coarse grained force field for ILs, that was extended for ILCs [46,47,48,49]. A comparison between the simulations and the experimental data available of a model ILC demonstrated that the model was qualitatively accurate. Nevertheless, the transition temperatures were wrongly predicted, especially the melting temperature *T*_m_. Running series of simulations with atomistic force fields, that are fully parameterized to reproduce structural and dynamic interactions on the SmA ionic mesophase, would mean a step towards a quantitative description [50], however the development of atomistic force fields is indeed a major challenge in the computational field. Overall, theoretical and simulation approaches are still in their infancy and progress in the field is mostly determined through experimental methods.

## 4. Materials Development in Ionic Liquid Crystals

ILCs are commonly built-up from long hydrophobic chains linked to a positively charged organic core. This structure favors the formation of smectic (lamellar) structures by micro-phase segregation of the charged and hydrophobic groups. Among a huge variety of ionic mesogens studied so far, the majority is derived from imidazolium cations. Other commonly investigated ILCs are based on ammonium, phosphonium, pyridinium, and more recently, viologens or guanidinium cations.

The research field during the early years focused on developing structure-properties relations to understand self-organization and the interactions that stabilize the ionic mesophase. Most of this work was centered around the investigation of the effect of the aliphatic chain length on the thermal behavior of these ionic compounds. Later, a considerable amount of work also focused on expanding the realm of accessible mesophase by ILCs, mostly by changing the number of aliphatic chains in the material. Systematic studies on the counter ion and the introduction of functional groups in the aliphatic chains lacked in these early days. The incorporation of (substituted) heterocyclic cores was, although synthetically more challenging, highly rewarding in the sense that mesophases were found at more accessible temperature ranges. These heterocyclic structures are still the workhorse to date and will be further discussed later in this section.

Research on the variation of the counter ions did, however, not develop to the same level as the work on the cationic moiety. Until 2012, the majority of the ionic mesogens synthesized contained a standard number of counter ions mainly of hydrophobic nature (BF_4_^−^, PF_6_^−^, NTf_2_^−^, I^−^). The counter ion provides an additional tool to efficiently modify the mesophase behavior of an ILC, but also other physical properties, including for instance viscosity [51]. To illustrate this, recent studies on the dimensional effects of anions (and cations) showed, in fact, that one should consider ILCs to be composed of “congruent ion pairs” rather than studying them independently [52]. Previous work did study the anion independently from the mesogenic cation, albeit the authors drew similar conclusions. This work highlighted the tremendous effect that the anion has on the physical properties of the material [40].

In order to review and summarize the key steps in the development of the field of ionic liquid crystals, we will discuss different classes of cations, starting from very simple soap-like aliphatic ammonium salts and ending with more complex mesogens where the cationic groups are incorporated into a more traditional mesogen.

### 4.1. Aliphatic Cations

Structurally the simplest ILCs are composed of a nitrogen or phosphorus atom substituted by one to four (long) aliphatic chains. In the case of amines, when less than four alkyl substituents are used a charged center can be generated by protonation of the nitrogen atom. The following sections discuss these straightforward ILCs, starting from protonated primary amines and continuing by alkyl-substituted ammonium and phosphonium salts. Because of the limited availability and the high chemical reactivity of the typical starting materials of phosphonium-based ILCs (trialkylphosphines) this class of materials was studied in less detail than the corresponding ammonium salts. As both materials do share the molecular design, they are discussed together. Despite the similarity in structure, the thermal behavior and mesogenic textures showed by these two cationic cores are remarkably different.

#### 4.1.1. Protonated Amines

**Figure 3:**
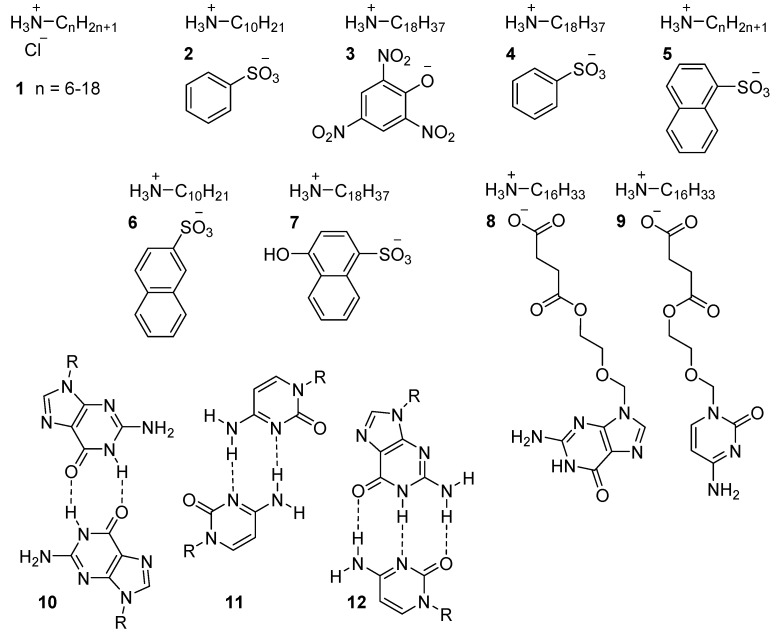
Protonated amine ILCs.

The simplest type of LC ammonium salts are based on *n*-alkylammonium chlorides (Figure 3) [53]. They are easily obtained by bubbling hydrogen chloride through a solution of *n*-alkylamines. In one example, the length of the alkyl chain substituted to the cationic moiety was studied [54]. Short alkyl chain compounds are too hygroscopic and their phase behavior was not described. The thermal behavior of the compounds with alkyl chain lengths *n* = 6–18 were investigated. These compounds showed SmA phases when heated above their melting points. The clearing temperatures were not clearly defined since the smectic domains coexisted with the isotropic liquid, pointing to decomposition.

These classical soap-like materials have great structural resemblance to the inverted analogues where the hydrophobic tail is attached to the anion and the salts are complimented with a small inorganic cation. Examples of these materials are alkali carboxylates (for instance sodium stearate) and alkali alkyl sulfonates [55,56]. Although LC phases have been observed for these materials, the transition temperatures are of the order of the decomposition temperatures, making analysis unreliable, in analogy to the ammonium salts discussed above. These materials, both the anion and the cation-based materials do form well-defined lyotropic phases.

To reduce the high transition temperatures of the ammonium chlorides, ionic salts bearing anionic moieties of an organic nature were synthesized and studied. This approach will come back multiple times in this manuscript. Matsunga and coworkers investigated the thermal behavior of ionic systems based on alkylamines and anions based on aromatic acids [57,58]. Since the (simple) cation is kept constant in these studies, this work is a clear example of the role of the counter ion influencing the mesomorphic behavior of the material. In the first approach, two series of alkylammonium benzenesulfonates **2** and picrates **3** were selected as target molecules and their thermotropic behavior was investigated. Nitrate and sulfonate groups are well known for their strong electron withdrawing effect on aromatic rings and thus, in this case, a stabilization of the negative charge on the anion was anticipated.

This study highlights the difference in the ability of these two anions to stabilize a mesophase. Picrates **3** displayed mesophases with cationic cores composed of aliphatic amines with carbon chain lengths of, at least, heptadecyl or octadecyl groups, whilst the benzenesulfonates **2** also showed mesomorphic behavior with much shorter alkylamines, from decyl to octadecyl alkyl chains. Furthermore, the thermal behavior of pyridine-3-sulfonate anion **4** showed a remarkable mesophase stabilization compared to that of compound **3**. This result was attributed to hydrogen bonding interactions between the ammonium cation and the nitrogen atom of the pyridine counter ion **4**. To further investigate the ionic interaction involved in the mesophase stabilization, this study was extended to anions based on fused aromatic rings naphthalene-1-sulfonates **5**, naphthalene-2-sulfonates **6** and 1-naphthol-4-sulfonates **7**. The higher *T*_m_ and *T*_c_ for **6** compared to **5** is explained by the increased degree of interdigitation of the 2-substituted naphthalene with respect to the 1-subsituted anion. Furthermore, the introduction of the OH group in **7** favors hydrogen-bonding interactions in the ionic system, resulting of a positive stabilization of the mesophase of compound **7**, reflected by an increase of both *T*_c_ and *T*_m_. No mesomorphic phase was observed for molecules with aliphatic tails shorter than thirteen carbon atoms attached to the cation. Studies on the influence of the tail length show that the mesophase widens markedly by increasing the chain length. These early results touch on some of the fundamental parameters of ILC design: chain length, anion design and the introduction of targeted interactions (dipolar, π–π, π–cation, hydrogen bonding, *etc*.), both in the anion or in the cation.

An example where the additional targeted interactions largely progress the field is the report on ILCs based on guanidine and cytosine [59]. In both materials, the mesogen is composed of a nucleobase, equipped with a short acid-functionalized tail that forms an ILC with an aliphatic amine. These types of salts formed smectic phases as a result of assembly at two levels: segregation of incompatible units (lipophilic to hydrophilic moieties) due to the formation of alkylammonium salts of **8** and **9** and dimerization [59]. Self-dimerization of these salts through molecular recognition resulted in homodimer formation towards base pairs guanine-guanine **10** or cytosine-cytosine **11**. Equimolar mixtures of guanine and cytosine salts resulted in heterodimer formation by molecular recognition **12**, which at high temperatures did exhibit smectic behavior.

More complex examples of this approach include the upcoming area of ionic liquid crystalline dendrimers in which the amine is not a simple aliphatic amine, but rather a amine terminated dendrimer [60,61,62,63]. The anions can be as straightforward as linear or branched carboxylic acids or complex and include entire mesogenic moieties. Because of their high molecular weight and associated properties, this class of materials is not further discussed in this manuscript.

#### 4.1.2. Quaternary Ammonium and Phosphonium Salts

In literature, quaternary ammonium salts are often compared to their equivalent phosphonium salts [3,4,64,65]. Both materials share a similar synthetic route: a quaternization reaction of trialkylphosphines or trialkylamines with an alkyl halide [54,66]. Due to the lower availability of the phosphonium salts, however, the phosphorous analogues of these ILCs have received less attention. Overall, the two quaternary salts differ in their mesomorphic behavior; phosphonium salts commonly display SmA_2_ mesophases, whereas the ammonium salts often show the more straightforward SmA phases [67]. In addition, phosphonium salts show a better stabilized LC phase, indicated by the wider thermal range of the mesophase with a notable increase of the clearing temperatures. Furthermore, they also show higher thermal stability compared to ammonium salts. The rationale behind the difference in thermal behavior observed for these materials is attributed to orbital levels: the 3*d* orbitals available in the phosphonium atoms and not in the second row nitrogen atoms participate in bonding interactions [68,69].

##### One Long Alkyl Chain

**Figure 4:**
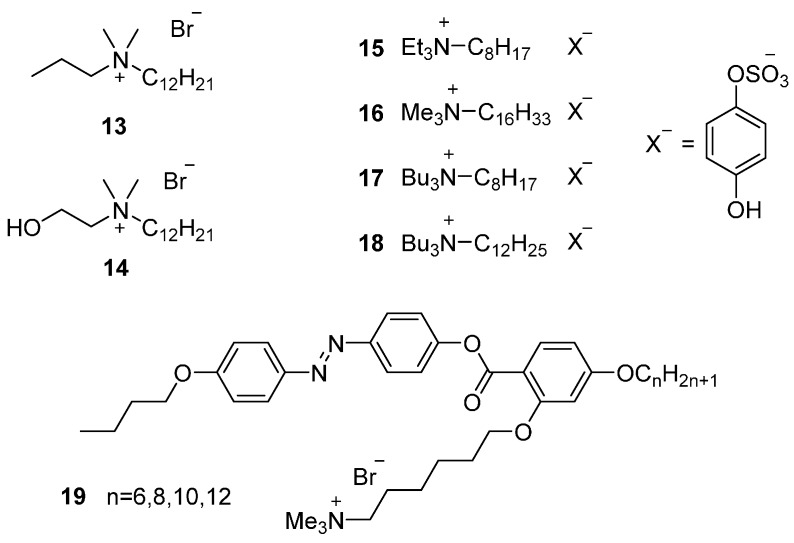
Ammonium-based ILCs with a single long aliphatic tail.

The first report, by Iwamoto and co-workers discussed the thermal behavior of quaternary ammonium salts containing a single long aliphatic chain with chloride, bromide or iodide as counter ion [70]. Due to the thermal decomposition of these materials at high temperatures, the studies could not verify a LC phase after the observed *T*_m_. Following studies were able to determine thermal properties of such mesomorphic materials by substituting one of the methyl groups by longer alkyl chains or short functionalized alkyl groups. The initial approaches were focused on studying the thermal behavior of different cationic cores, whilst keeping the same anionic moiety bromide. Both propyl (Figure 4, **13**) and hydroxypropyl compounds **14** showed a SmA phase. Compound **14**, however showed a wider thermal range for the mesophase given by a remarkable increase of *T*_c_. This result highlights again the role of hydrogen bonding as a tool to stabilize mesophases. The work was extended to other functional substituents (alcohols, carboxylic acids, cyano groups) [71,72,73,74,75] including a terminal double bond [76]. The overall conclusions from these studies show that the introduction of different polar groups on aliphatic chains causes changes of the micellar parameters similar to those changes caused by increasing the length of the alkyl chain. The thermal stability and the range and number of the textures are comparable, but intrinsically dependent on the nature of the functional group introduced.

As with the protonated materials, ILCs with anions with a more organic character, are sometimes, more stable and easier to manipulate. This allows then, to investigate the substitution pattern on the cationic core of the mesogens. The triethyloctylammonium **15** and trimethylhexadecylammonium **16** cations show a SmA phase at room temperature and 80 °C, respectively. The corresponding bulkier substituted tributyloctyl **17** and tributyldodecylammonium **18** salts, however, are not mesomorphic. The work suggests a different approach of manipulating the thermal behavior of the material by considering the ionic moieties as counter ion pairs. A proper balance of the size and interactions of both ionic moieties would lead to desired properties like room temperature mesophases [3].

An important contribution that should be mentioned features a calamitic azobenzene-based mesogen with a quaternary ammonium group attached laterally through a short spacer **19** [15]. Depending on the length of the terminal alkyl chain the authors observed different mesophases. The mesogens containing C6 or C8 terminal tails showed the highly atypical nematic phase close to room temperature (both *T*_m_ and *T*_c_). When longer side alkyl chains were introduced, C10 and C12, the nematic phase was displaced by SmA phase. This work is the only example of an ILC displaying a room temperature nematic phase until today, despite the significant efforts by multiple research groups to find similar or alternative structures with the same properties. The general architecture of these molecules where a more traditional mesogen is combined with an ionic group, either with or without a flexible spacer is discussed in detail later in this work.

Phosphonium salts, bearing one, two or three long alkyl chains, were studied by Weiss and coworkers [67,77,78]. The phosphonium salts with one long alkyl chain displayed SmA_2_ phases characterized by a strong interdigitation between alkyl chain and therefore higher thermal stability and mesomorphic windows; the phosphonium mesogens with multiple tails are discussed in the next paragraphs.

##### Two Long Alkyl Chains

**Figure 5:**
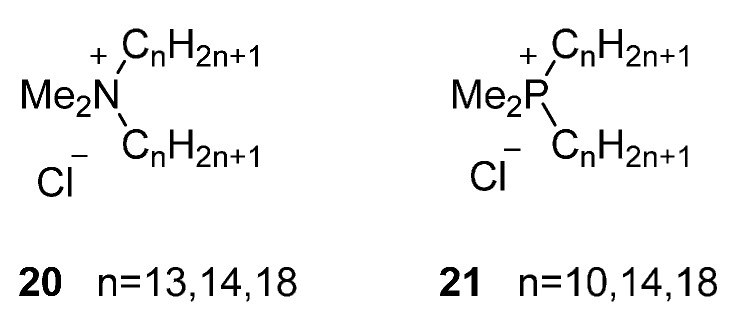
Ammonium and phosphonium-based ILCs with two long aliphatic tails.

For mesomorphic ammonium salts containing two long alkyl chains a new type of smectic phase, the SmT phase was found. The first example of quaternary ammonium salt displaying a SmT phase was found in *N*,*N*-dialkyl-*N*,*N*-dimethylammonium bromides (Figure 5, **20**) [14,79,80]. To display such mesophase, it is not necessary that the two long alkyl chains contain the same number of carbons. The molecular structure of the SmT phase, can be described by the ammonium head groups and counter ions packed into tetragonal vertex separated by each other by randomly oriented long alkyl chains (see also Figure 3).

By modifying the molecular design of quaternary ammonium salts with different functional groups, the SmT phase can be either completely displaced or can coexist with other mesophases. For instance, when a cyano group is present at the terminal position of one of the alkyl chains, the SmT phase is displaced by a SmA phase [72,74]. Furthermore, the SmT phase was found at lower temperatures than a SmA by Skoulios and coworkers in systems bearing 2-hydroxyethyl chains at the polar head group [81]. The homologous equivalent (*N*,*N*-di-*n*-alkyl-*N*,*N*-dimethylammonium bromide) with longer C14 and C18 tails do, however, not show SmT phases, but rather SmA_2_ phases where the tails of the mesogens show significantly less interdigitation than in the SmT phase and also than the tails in the ammonium salts bearing one long alkyl chain [82].

Kanazawa and coworkers studied the mesophase behavior of the corresponding phosphonium salts *N*,*N*-di-*n*-alkyl-*N*,*N*-dimethyl phosphonium chlorides (**21**) with long alkyl chains of C10, C14 and C18 [83]. Compared to the thermotropic ammonium salts with similar substitution patterns previously discussed, all the phosphonium salts exhibited a SmA phase. The mesophases of the phosphonium salts showed a wider thermal range and higher thermal stability compared to the ammonium salts.

##### Three or Four Long Alkyl Chains

**Figure 6:**
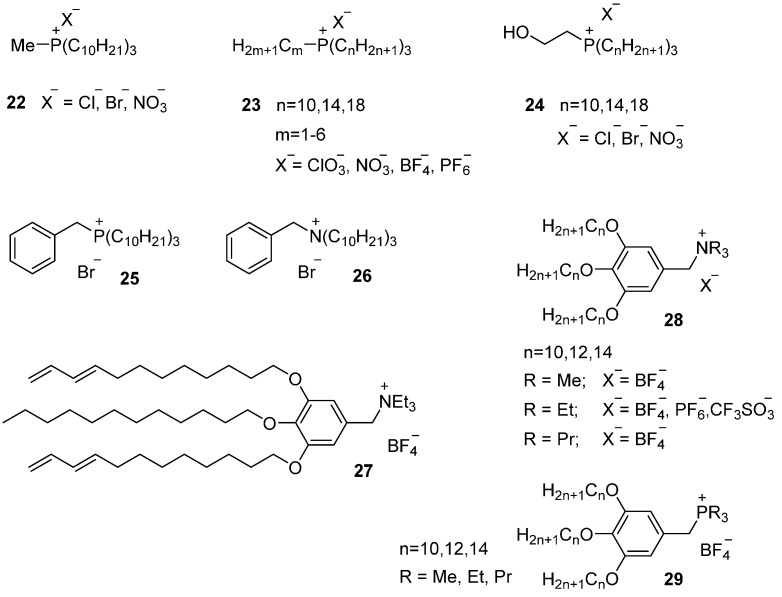
Ammonium and phosphonium-based ILCs with three or four aliphatic tails that are either substituted directly to the cation (**22**–**26**) or connected through an aromatic spacer (**27**–**29**).

Weiss and coworkers investigated the mesophase formation of phosphonium salts containing three or four long alkyl chains [67]. Methyl tri-*n*-decylphosphonium chloride and bromide (Figure 6, **22**) just showed a crystal phase, but their monohydrate versions behave as a room temperature ionic liquid crystal showing a SmA_2_ phase. The reason for the destabilization of the crystal phase was mainly attributed to the hydrogen bonding between the halogen anions and the water molecules. When the anionic moiety of hydrate version was exchanged by a nitrate counter ion, for instance, the crystals phases were followed by a mesophase at around 60 °C. By adding a small amount of acetonitrile (*ca.* 1 wt %), the transition temperatures dropped dramatically and melting points became sub-ambient. This thermal behavior pointed to the formation of liquid-crystalline clathrates due to the presence of acetonitrile. Later studies remark more extensively the ability of (thermotropic) phosphonium salts **23** to change their thermal behavior by the addition of a (small) amount of a proper protic solvent (water, methanol) [77,84], or by attaching hydroxyl groups to the mesogen **24** [85]. The aggregation equilibria of tri-*n*-dodecylammonium chloride and nitrate in benzene was also investigated [86].

Benzyl-tri-*n*-octadecylphosphnium bromide **25** is an example of a phosphonium salt that contains four large chains. The mesomorphic behavior of this material was characterized by a SmA_2_ phase. Its thermal stability is higher than its analogous ammonium salt **26**, which shows, however, a wider mesomorphic window than the phosphonium salt [66,87]. No mesophases were observed for symmetrical tetra-*n*-alkylphosphonium halides.

A special class of thermotropic materials based on quaternary ammonium salts form so-called biscontinuous cubic liquid crystals (Cub_bis_) [88,89,90]. These materials were used in the preparation of ion conductive polymeric films with three-dimensional interconnected ion channels. The molecular design of these mesomorphic compounds was based on wedge-shaped ammonium salts (based on ionophobic and ionophilic parts). The molecules **27** were based on polymerizable ammonium moieties complexed with a lithium salt. Thermal studies of the monomer indicated the formation of the Cub_bis_ phase between −5 and 19 °C on a heating ramp and a hexagonal columnar phase (CoI_h_) at higher temperatures, 19–56 °C. The Cub_bis_ organization can be preserved by an *in situ* photo-polymerization reaction leading to a free-standing and optically transparent film. Polymer films with Cub_bis_ LC nanostructures exhibit higher ion conductivities than films obtain by photo-polymerization in the CoI_h_ or isotropic phase and the three-dimensional interconnected ion channels derived from the Cub_bis_ phase were considered as efficient ion conductive pathways.

In a later work on Cub_bis_ LC materials, phosphonium salts were studied for comparison with the equivalent ammonium salts. Focused on the promising wedge-shaped molecular design, two series of ammonium **28** and phosphonium **29** salts were prepared and there mesomorphic and ion-conductive properties were investigated [91]. Ammonium and phosphonium salts showed similar phase transition behavior. These ionic salts induce mesomorphic phases (Cub_bis_, CoI_h_) depending on the chemical structure of the ionic moiety, the length of the alkyl chain or the type of counter ion. Furthermore, phosphonium salts exhibiting Cub_bis_ mesophases showed higher ionic conductivities at room temperature than the homologous ammonium-based Cub_bis_ LC salts.

##### Aliphatic Heterocyclic Cations

For ionic liquid crystals based on heterocycles different physical properties were expected. Firstly, the ionic group is more shielded by the aliphatic rings, which impacts the micro-phase-segregation between the ionic and nonpolar parts that forms the basis of phase formation. Secondly, by using aromatic heterocycles, the charge density is spread out over a much larger volume than is the case in the straightforward ammonium salts, which similarly effects the mesophase behavior [92].

**Figure 7:**
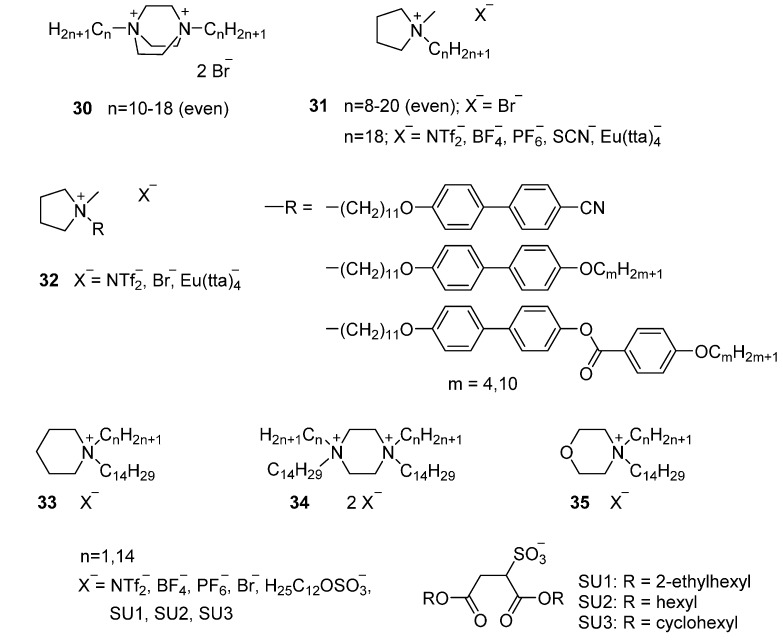
ILCs based on aliphatic heterocyclic cations (**30**–**33**,**35**) and dications (**34**), substituted with one or more aliphatic tails or with classical thermotropic mesogens.

Chronologically, among the first cyclic (aliphatic) ammonium salts studied were 1,4-diazoniabicyclo[2,2,2]octane dibromides (Figure 7, **30**) [79]. These mesomorphic di-cations with different chain lengths exhibited SmT phases. A valuable feature of this material is that it shows a strong tendency to spontaneously align homeotropically, not just during the cooling process from the isotropic phase, but also during the heating ramp.

Pyrrolidinium bromide salts **31** exhibit mesophases with alkyl chains longer than C11 tails [93]. The bromide counter ion can easily be exchanged by a wide range of anionic species with a different nature (hydrophobic, metal coordinated, *etc*.). Pyrrolidinium compounds exhibit a rich polymorphism (SmT, SmE, disordered SmA and hexagonal columnar phases). The length of the alkyl chain and the nature of counter ion have a great influence on the mesomorphic behavior of the final material. An increase of the aliphatic tail length stabilizes the mesophase slightly. The authors attributed the differences in thermal behavior to the different size of the counter ion. The compounds with BF_4_^−^, PF_6_^−^ or SCN^−^ anions have similar sizes, explaining their similar behavior. The absence of a mesophase in NTf_2_^−^ and europium-based salts was attributed to the larger size of these anionic species.

The important role of the counter ion to influence the thermal properties of the material is also underlined by the pyrrolidinium-based mesogens [94]. In this work different pendant biphenyl-derived groups were linked to the pyrrolidinium cationic core via a flexible alkyl spacer **32**. With the exception of the ionic salts containing NTf_2_^−^ counter ions the materials showed rich polymorphism of highly ordered smectic phases. As expected, salts with longer spacers and terminal alkyl chains showed lower melting points than their shorter homologues. Again, the europium containing salts did not show mesophases, attributed to the bulkiness of the counter ion that prevents mesophase formation.

In addition, researchers studied the mesomorphic behavior of ionic salts based on different aliphatic heterocycles, such as piperidinium compound **33**, piperazinium compound **34** and morpholium compound **35**. The central goal of these studies was to develop room temperature ionic liquid crystals [92]. From a molecular design point of view, all these cationic cores contain six-membered rings and a localized positive charge. Most ionic liquid crystals studied (imidazolium and pyridinium salts) are charge delocalized cationic cores only display SmA phases. Therefore, aliphatic heterocycle systems open the possibility to explore whether ionic liquid crystals bearing a localized charge exhibit unusual mesomorphic behavior [95]. The notable polymorphic nature of the latter materials was attributed to the nature of the cationic core. The most remarkable mesomorphic behavior was reported for the morpholinium sulfosuccinate salts. These materials showed room temperature hexagonal columnar mesophases with clearing points around 120–147 °C. X-ray diffraction analysis and molecular modeling confirmed the unexpected hexagonal columnar phase: Via a self-assembly process of three molecules; a disc-shaped supramolecular mesogen is obtained, that subsequently orders into a hexagonal columnar phase. The piperazinium-based compounds **34** that solely form LC phases with the sulfosuccinate anions, showed different mesomorphic textures (SmA, SmE or SmT at temperatures below 100 °C). Studies on piperidinium core showed that a minimum alkyl chain of 14 carbon atoms was necessary to induce mesomorphic behavior.

Although the authors aimed to decrease transition temperatures by changing the cationic core, the largest effects were obtained by modifying the counter ion. A dramatic decrease on the melting point of piperidinium, piperazinium and morpholium salts was realized by replacing the chloride anions with anionic moieties such as dodecylsulfate (DOS) or different dialkylsulfosuccinates (SU). The identification of room temperature ionic liquid crystals is important for future applications. In the case of ionic liquid crystals showing columnar phases, this would lead to anisotropic ion conductivity, as such materials are expected to show higher conductivity along the columnar axis rather than perpendicular to it [34].

### 4.2. Aromatic Cations

**Figure 8:**
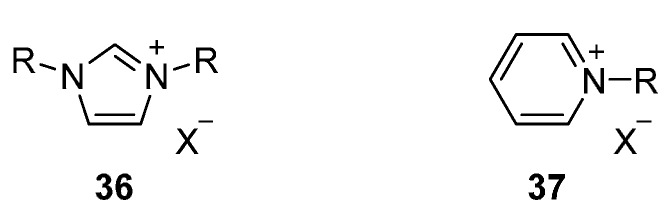
Generic structure of the two most investigated classes of ILCs: imidazolium (**36**) and pyridinium (**37**) salts. The imidazolium salts have the advantage that they allow easy asymmetric substitution at the heteroatoms of the ring.

Ionic liquid crystals based on aromatic cationic cores have received most attention in the field. The major difference with the aliphatic cations discussed above is the charge distribution of the cation over a larger volume. This reduces ionic interactions, yielding materials with lower transition temperatures and therefore a better application potential. Different examples of such aromatic ILCs, based on for instance imidazolium, pyridinium, guanidinium, quinolinium and viologen have been reported [4,41,50,96,97,98,99,100]. Due to their good accessibility and availability for synthetic manipulation, some of them have been studied and exploited to a great extent over the years. Much of the work on ILCs (and in fact, also much of the work on ILs) focuses on two aromatic cores: imidazolium (Figure 8, **36**) and piridinium **37** cationic cores.

Qualitatively, imidazolium and pyridinium salts show some similarities that explain their popularity in the field. From a synthetic point of view, the excellent (commercial) availability of the starting materials and the relatively high reactivity given by electrophilic nitrogen centers allow access to a large library of ILCs where structure-property relations can be studied in great detail. The driving forces for the formation of pyridinium and imidazolium liquid crystals are similar: ionic, dipole-dipole and Van der Waals interactions of the aliphatic tails (analogous to the aliphatic ILCs) in addition to π–π and π–cation interactions from the aromatic core.

There are, however, also some differences that limited the work on pyridinium salts compared to the imidazolium salts in the past years. The imidazolium salts contain two electrophilic centers, which doubles the possibilities for functionalization and the introduction of different moieties and tailor the (thermal) properties of the mesogen. In pyridinium salts, this is synthetically more cumbersome to realize. In addition, the five-membered ring of the imidazolium core leads to more bend-like molecules compare to the linear *para*-substituted phenyl group, associated to higher melting and clearing transition temperatures for pyridinium than for imidazolium salts [101].

In the following paragraphs, we will separately discuss the thermal behavior of these two types of aromatic ILCs; with simple aliphatic tails and with core-extended groups, that brings them closer to classical non-charged mesogens. Due to the advantageous thermal behavior displayed by the imidazolium salts, we will focus our attention on these materials and only mention some examples of pyridinium-based ILCs in the first paragraphs.

#### 4.2.1. Simple Aliphatic Tails

In analogy to the quaternary ammonium salts, discussed earlier, the simplest types of aromatic ionic salts have a charged, delocalized core that is substituted by one or more alkyl chains. Typically, such materials display SmA phases [101,102,103], where the molecules are arranged in layers that are stabilized by different intermolecular interactions (but dominated by ionic interactions).

**Figure 9:**
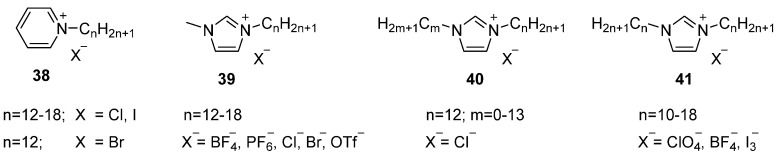
Pyridinium (**38**) and imidazolium-based (**39**–**41**) ILCs with one or two long aliphatic tails.

Imidazolium and pyridinium ILCs with a single substituted long alkyl chain (Figure 9) show qualitatively common structure-thermal property relations: Firstly, the temperature range of the mesophase increases with increasing the alkyl chain length. The minimum chain length to display a mesomorphic phase, however, depends on the anionic moiety of the ILC [101,104,105,106,107,108]. The introduction of different counter ions in the same cationic moiety has a higher impact on *T*_c_ than on *T*_m_, similar to what was found for earlier discussed salts [57,58]. In the case of *N*-(*n*-alkyl)-pyridinium halides (Figure 9, **38**) the observed temperature range decreases in the order Cl^−^ > Br^−^ > I^−^ [107,108]. The influence of the anion on the mesomorphic behavior of 1-(*n*-alkyl)-3-methyl-imidazolium salts (*n* = 12–18) with PF_6_^−^ and BF_4_^−^ was investigated in different studies **39** [106,109]. Both series showed a SmA phase, however, the imidazolium salts bearing BF_4_^−^ anions displayed a mesophase already with dodecyl chains (*n* = 12). The homologues ionic salts bearing PF_6_^−^ as counter ion displayed mesomorphic behavior with tetradecyl chain (*n* = 14) and, in general show higher melting temperatures than the ionic salts with the BF_4_^−^ counter ions. In a related study, a series of 1-(*n*-alkyl)-3-methyl-imidazolium, containing Cl^−^, Br^−^, triflate (OTf^−^), BF_4_^−^ and bis(trifluoromethanesulfonyl) imide (NTf_2_^−^) as counter ions was investigated [102]. All the BF_4_^−^, Cl^−^ and Br^−^ salts investigated bearing a minimum alkyl chain of *n* = 12 exhibited SmA_2_ phases. The studies attributed two factors to the interlayer spacing dependency of the mesomorphic materials: (i) The interlayer spacing increases by increasing the length of the alkyl chain; and (ii) The interlayer spacing strongly depends on the nature of the anionic moiety and increases in the order OTf^−^ < BF_4_^−^ < Br^−^ < Cl^−^ [102]. In other words, this order is in agreement with the ability of the counter ions to form 3D hydrogen-bonding lattice. However, studies also point that thermal behavior of hygroscopic counter ions (like Cl^−^ and Br^−^) are more complicated than the more hydrophobic anionic moieties (like PF_6_^−^ or BF_4_^−^). The main difference is attributed to the difficulty to obtain those ionic salts bearing hydrophilic counter ions in the anhydrous form. Anhydrous salts have lower melting points and enthalpies than hydrated ones. This is attributed to the fact that the structure of the hydrated salts is stabilized by hydrogen bonding between the hydrophilic anion and the water molecules. This results indicates that previous chloride salts studied by the same author were indeed hydrated [101]. A detailed study on the long-chain 1-(*n*-alkyl)-3-methyl-imidazolium chloride attributes the thermal behavior of the hydrated chloride salts to conformational changes on the imidazolium moieties [104].

The imidazolium core, pre-eminently is suited for the introduction of two the same or different aliphatic substituents [103,110,111]. The symmetrical substitution pattern enhances their rod-shaped character and promotes the lower ordered monolayer SmA phases over the bilayered SmA_2_ phases that are often found for more strongly asymmetric mesogens **40**. Mesogens with two C10 or longer alkyl chains **41** (and their mixtures) show SmA phases with temperature windows strongly dependent on their counter ion. Recent work showed that room temperature mesophases are in reach for these straightforward mesogens equipped with BF_4_^−^ or ClO_4_^−^ anions [112]. Recent work also demonstrates the advantages of using such compounds (**41**, *n* = 12, X^−^ = I_3_^−^) as anisotropic electrolytes in dye-sensitized solar cells [113].

**Figure 10:**
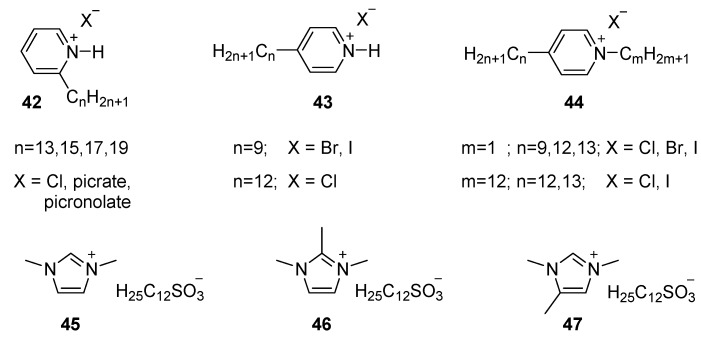
Pyridinium (**42**–**44**) and imidazolium-based (**45**–**47**) ILC with alternative substitution patterns show that in some cases, small changes in the cation structure are very important for the physical properties, while in other cases small structural changes are allowed.

The geometry and substituent structure of the cation plays an important role in the phase formation of such materials. For instance, in protonated alkylpyridines (Figure 10, **42** and **43**) mesophase formation depends on the relative position of the ammonium center and the aliphatic chain. A LC phase is observed for **42** with the chain close to the ammonium center [107], but not for **43** with the chain opposite to the cationic center. Methylation of the nitrogen atom, however did induce mesomorphic behavior on the materials **44** (*m* = 1) [114]. Alkylation of the nitrogen atom again induces mesomorphism on the material, even when the side chain is placed on the 4 position **44** (*m* = 12) [115]. This material has two tails and, hence is more likely to display liquid crystalline properties, as it is discussed in the following paragraphs.

Another example, but following a slightly different approach, is given by imidazolium salts where the long alkyl chain was placed on the anionic, moiety rather than on the cationic core. Experimental evidence from other studies indicate that this change does not bear a large effect on the mesomorphic properties, as the ion pair should be considered as a pair rather than the individual components [40,41]. It was observed that 1,3-dimethyl imidazolium dodecylsulfonate **45** displayed a SmA phase. A methyl group on the imidazolium 2-position **46** suppresses the mesophase, whilst substitution on the 4-position **47** effectively suppressed the crystallization and opened the temperature gap for mesophases [116].

**Figure 11:**
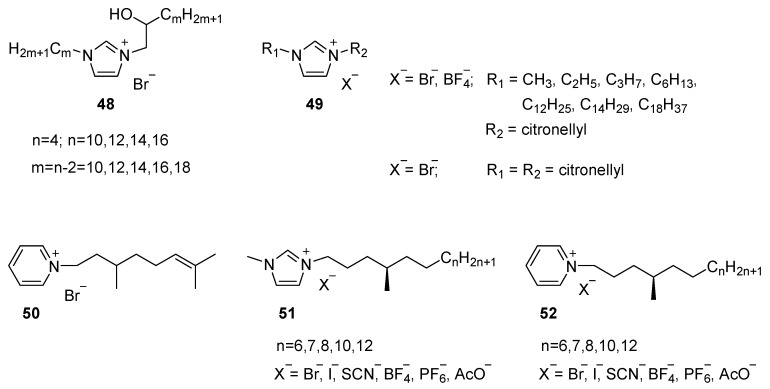
Substituents at the heteroatoms of imidazolium (**48**,**49**,**51**) and pyridinium (**50**,**52**) ILCs.

Alternatively, functional groups can be introduced in the aliphatic tails. Analogous to what was observed in the ammonium salts, hydroxyl groups in the β-position from the imidazolium core widen the temperature range of the mesophase. Mesomorphic materials experienced a decrease of the melting point (many of them below room temperature) as well as an increase of the clearing temperature (Figure 11, **48**) [117]. The ion-dipole and hydrogen bond interactions in these mesogens effectively increase the rigid core size and stabilize the hydrophobic side chain organization in the layered mesophase over a wide range of temperature. At the same time, the steric hindrance induced by the (racemic) hydroxyl group suppresses crystallization by allowing molecular rearrangement inside the LC phase until sub-ambient temperatures.

The introduction of a methyl group in the aliphatic tail (**49** and **50**) generating a tertiary (chiral) carbon atom, also introduces steric effects but lack the stabilizing interactions [118]. Mesomorphic behavior was just observed for imidazolium salts bearing a tetradecyl hydrocarbon chain and bromide as counter ion and in compound **50**. Imidazolium **51** and pyridinium **52** salts containing a methyl group in position four (chiral) to the nitrogen atom were investigated in later studies [119]. Of particular interest in this work was to explore the effect of the counter ion on the mesomorphic behavior of the target ionic salts. All mesogenic materials reported in this study displayed a SmA phase. Their mesophase stability decreased in order Br^−^ > AcO^−^ > I^−^ > BF_4_^−^ > SCN^−^ > PF_6_^−^ for the imidazolium salt (**51**) and Br^−^ > AcO^−^ > BF_4_^−^ > I > SCN for pyridinium ILCs **52**. Overall however, the pyridinium mesogens showed much lower transition temperatures compared to the imidazolium salts.

#### 4.2.2. Mesogenic Moieties

Besides the introduction of long (branched) aliphatic chains, the liquid crystal properties of ILCs may be modified by extending the rigid aromatic part of the mesogen. As a general trend, the incorporation of a phenyl group to an ionic salt stabilizes the liquid crystal phase. Consequently, the temperature range shown by these materials, are much narrower than those previously discussed, composed of imidazolium or pyridinium cores bearing alkyl chains. The presence of an additional phenyl group in the core has several pronounced effects: firstly, the geometric increase of the rigid part of the molecule simply increases the transition temperatures, in particular the melting temperature. Secondly, the aromatic group increases the electron density of the core, favoring cation–π and π–π interactions; contributing both to stabilize order in the material. Lastly, when an aromatic ring is directly substituted to the cation, it increases the π-delocalization of the cation (in particular in the case of *N*-arylimidazoles that adopt a nearly flat conformation that again, stabilizes the packing of the mesogens). On the other hand, the increased of electron density positively impacts the bulk optical properties (birefringence) of the material, that is typically very low for the (di)alkylimidazolium or pyridinium ILCs and even lower for the corresponding ammonium salts.

The optical properties may be further improved by functionalizing the aromatic ring with electron donating substituents. In addition, the combination of these electron-rich core extensions and the electron deficient cationic pyridinium or imidazolium ring can induce a large dipole moment, which also contributes to stabilization of the (meso)phases. The combined effects are particularly strong in pyridinium-based ILCs, that show both high *T*_m_s and *T*_c_s, and as a consequence, pyridinium-based structures have been considered less than their imidazolium counterparts for practical applications and were, therefore, much less studied in the past years. ILCs with extended imidazolium-based cores are the topic of the following paragraphs. The discussion is based on the three different cases of core extension: (i) the case where the mesogen and the aromatic ring are separated by a small (aliphatic) spacer; (ii) where they are directly attached; and (iii) where they are separated by a long aliphatic spacer that, effectively, decouples both moieties.

**Figure 12:**
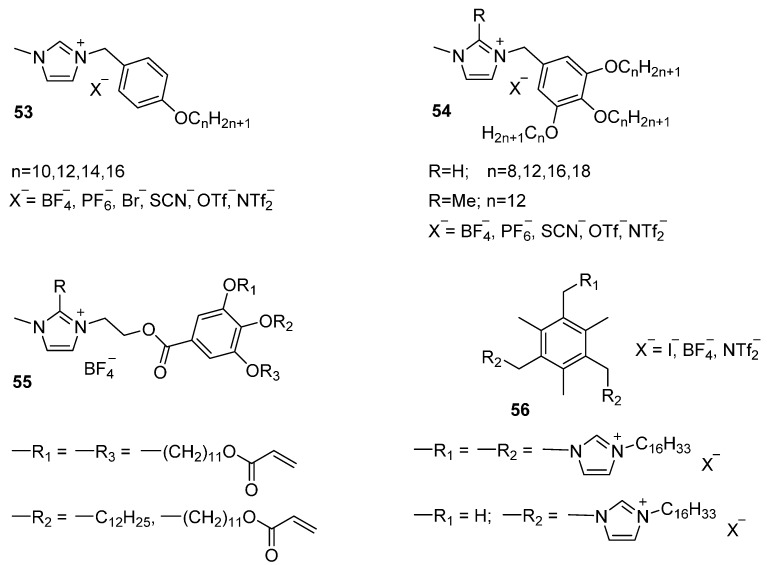
Imidazolium-based ILCs conjugated to aromatic groups bridged by a short aliphatic spacer. The number of tails, as well as the number of cationic cores is readily modified in this approach.

Calamitic mesogens with a methylene spacer (Figure 12, **53**) are obtained via a simple synthetic route [120]. A readily accessible starting material like a benzyl halide functionalized in the *para* position with a long aliphatic alkoxy group, reacts smoothly with an imidazole precursor, yielding the ILCs in nearly quantitative yields. Series of benzyl-substituted ILCs with different counter ions were prepared by a simple counter ion exchange step based on a metathesis reaction. These mesogens show SmA phases between low melting temperatures and high clearing temperatures. The authors point to a clear trend in phase stabilization by decreasing the size of the counter ion (Br^−^ > BF_4_^−^ > SCN^─^ > PF_6_^−^). Although the same trend was already observed for ILCs previously discussed, it may be an oversimplification to only attribute this to anion size. Furthermore, in the same study the effect of manipulating the length on the aliphatic alkoxy group to compensate the conformational freedom on the benzyl group was also investigated. Experimental results showed indeed an increase of the melting and clearing temperatures of the mesomorphic materials by increasing the chain length.The straightforward benzyl-based synthetic strategy also allows access to mesogens that promote columnar phases, interesting for anisotropic conductivity. By starting from benzyl halides with multiple alkoxy tails **54,** the formed wedge-shaped molecules self-organize into columnar assemblies that in turn, form columnar hexagonal mesophases. An increase of the Cr-CoI_h_ transition temperature was observed for an increased alkyl chain length [121]. Taking a step further, the ends of the aliphatic tails may be functionalized by polymerizable acrylate moieties **55** [122]. The self-assembled liquid crystalline structure can now be stabilized by photopolymerization in the liquid crystalline phase. Subsequent heating and cooling of the sample prevents phase transitions to occur, providing thermal stability for the resulting anisotropic ion-conductive films. An alternative approach towards discotic mesogens is to design a central aromatic core to which multiple imidazole groups can react. In this way, discotic molecules based on a mesitylene core **56** were synthesized [123]. By this approach, the aliphatic tails providing liquid crystallinity were introduced at the 3-position of the imidazole group. Despite their disc-shape, SmA phases over a wide thermal range and with low melting temperatures were reported for these materials. When the counter ion was exchanged to NTf_2_^−^, the material tended to supercool and crystallization was suppressed.

The easy synthetic procedures of the benzyl core, provides an excellent access to a wide range of materials, but its disadvantage is the highly bent conformation of the mesogen. A more natural solution is to remove the spacer and directly link the aromatic ring to the imidazolium (or pyridinium) cation. This logical approach was much less studied because of the more challenging synthesis of these molecules. Two different methods have been used for the synthesis of arylimidazolium ILCs. The first is based on the direct imidazolium ring-closure reaction of anilines [124] with glyoxal and a C1 reagent such as formic acid or a chloromethyl ester. Although this procedure has been used to generate asymmetric ILs [125] (and ILCs), it is much more convenient to use it for the synthesis of symmetric mesogens. The second method is the direct coupling of an aryl halide with a preformed imidazole [126]. The much poorer reactivity of arylhalide starting material compared to their benzylic and aliphatic homologues makes the latter route experimentally more challenging. Fortunately, progress in the field based on metal catalyzed cross-coupling reactions in the past decade improved the reactivity of imidazoles with phenyliodides and bromides [127,128,129], albeit relatively harsh conditions are still required, which impede the introduction of sensitive functional groups. The ILCs prepared by this route are asymmetric, as this reaction can only take place on the secondary nitrogen of the imidazole. A subsequent conversion to the imidazolium group should be realized with aliphatic nucleophiles [40].

In general, the arylimidazolium (ArIm) or arylimidazoliumaryl (ArImAr) mesogens give rise to relatively more rigid and linear cationic cores and follow more closely the design principles of ‘traditional’ non-charged mesogens. The materials properties are qualitatively comparable to the ILCs discussed previously: SmA phases over a wide thermal range are observed, but typically with high melting and clearing temperatures that further increase with increasing chain length. The high stability of the crystalline and SmA phases is directly related to the increased intermolecular interactions discussed previously. In the presence of highly polarized anions, however, the clearing point (and thus the mesophase stability) can be remarkably decrease; this class of ILCs was found to be significantly more dependent on the nature of the counter ion than other ionic mesogens [40,123].

**Figure 13:**
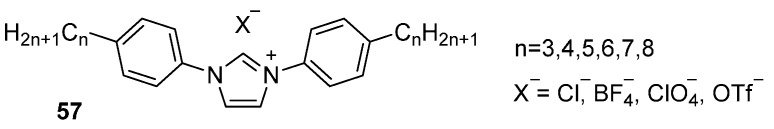
Generic structure of symmetric ArImAr-based ILCs with short aliphatic tails.

The ArImAr mesogens combine the extended aromatic core with a bend induced by the five-membered imidazolium ring. The first report on this class of ILCs used the two-step *in situ* imidazolium formation. In the first step two anilines reacted with glyoxal to form a glyoxal diimine, which in the second step was ring-closed in the presence of chloromethyl pivolate and AgOTf [130]. The mesogens (Figure 13, **57**) showed SmA phases with high transition temperatures and a small dependence of the clearing temperature with the tail length.

**Figure 14:**
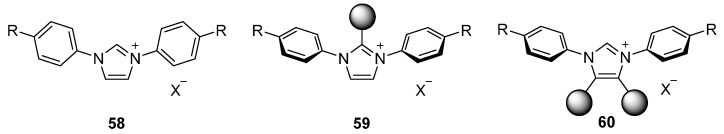
ArImAr mesogens **58** take a nearly flat conformation, perfect for (undesired) lateral mesogens-mesogen interactions. Introduction of small substituents on the 2 position or the 4 and/or 5 position of the imidazolium ring (**59**) forces the aryl rings to twist away from the imidazolium plane and reduces the interactions. In addition, phenyl-substitution (**60**) may give similar results.

In a quest for nematic phases in ILCs, we investigated an analogous synthetic route to generate these symmetric imidazolium salts, starting from commercially available anilines with short alkyl chains (C3–C8) instead of the long alkoxy substituents [102,108]. For the smallest tails, only crystalline materials were found, independent of the counter ion. Longer alkyl tails (C5–C8) gave SmA phases, where the widest mesophase ranges (and the highest transition temperatures) were persistently found for the materials with chloride anions [131]. A suggestion to reduce the strong Coulombic interactions that lead to the high transition temperatures is to prevent stacking of the aromatic cores by introducing small substituents on the imidazolium core (Figure 14). Substituents on the 2 position (**59**) as well as on the 4 and 5 position (**60**) will enhance the steric hindrance between the rings, forcing them to be placed out of the plane and hence, hindering stacking (Figure 14). The imidazolium ring closure procedure that we followed (an optimized modification from literature) does, however, not allow for small methyl-substituents on the imidazolium group. Alternatively, one should be able to introduce substituents at the *ortho*-positions of the substituted phenyl groups (**60**) with a similar effect.

**Figure 15:**
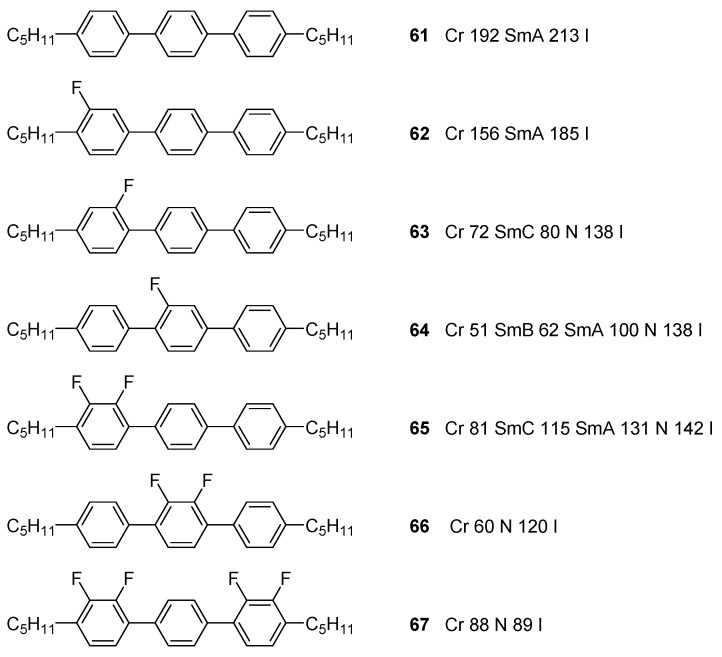
Influence of fluorine substituents on the phase behavior of terphenyl mesogens. Whereas the core terphenyl shows a smectic phase, the layer formation can be suppressed using fluoro-substituents. The most effective position is either in the central ring **66**, or on the *ortho* positions of the outer rings **67**.

Another well-known tool in the LC field to reduce packing efficiency of biphenyl or terphenyl mesogens is the introduction of lateral fluoro-substituents. The effects of this approach is beautifully demonstrated by the terphenyl series shown in Figure 15 [132]. The slightly larger size of fluorine compared to hydrogen atoms enhance intramolecular steric hindrance and increase inter-annular twist between aromatic rings in the core. This twisting lowers intermolecular π–π interactions and consequently, the melting points of the material. Due to low polarizability of fluorine, the substituents are preferably placed as lateral substituents. Larger polar groups, such as cyano-groups, are preferred as terminal substituents.

**Figure 16:**
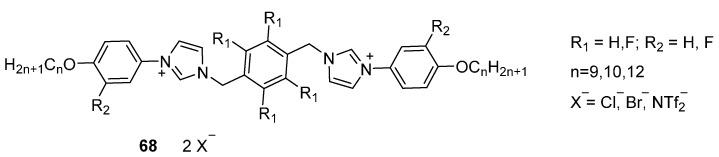
Di-imidazolium-based ILC to study steric ring effects.

So far, experimental studies that transfer this knowledge to ionic liquid crystals are limited. One example (Figure 16, **68**) is a mesogen with two phenylimidazolium moieties bridged by a phenyl core through methylene spacers [133]. The study aimed to investigate the role of fluorination on the central phenyl group as well as on the ArIm part. The effect of the latter, which is the most interesting for the field of ILCs to reduce transition temperatures is ambiguous from this study.

**Figure 17:**
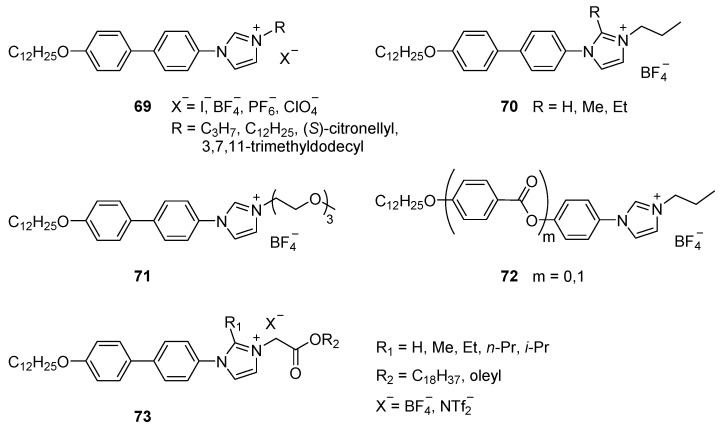
Structure of rigid-core Ar-Im imidazolium-based ILCs with different tails, cores and lateral substituents to tailor the thermal properties of the mesogens. In this class of ILCs, the cation is directly attached to an aromatic core which gives the molecules the structure and characteristics more in line with traditional mesogens.

The second route to ArIm ILCs uses an amination of aryl halides with imidazoles and yields asymmetrically substituted imidazolium salts [40,51,126]. The synthesis allows for substituents on the imidazole 2-position, although the rate of the cross-coupling reaction quickly decreases with the bulkiness of the substituent. In this way a wide range of aryl imidazole precursors (all with one alkoxy tail) were prepared and subsequently alkylated with different size and nature tails. Ion exchange disclosed the role of the counter ion in this series (Figure 17, **69**–**73**).

**Figure 18:**
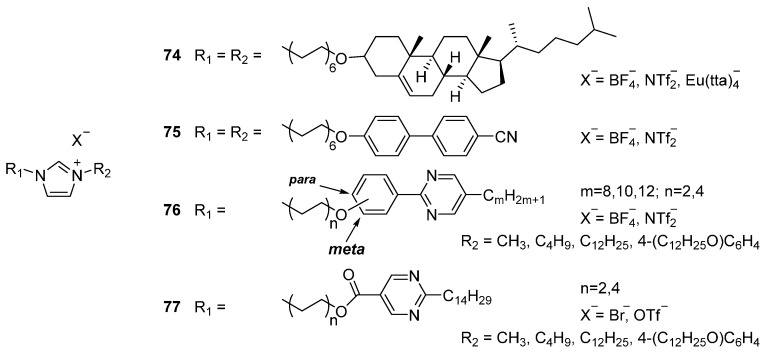
Imidazolium-based ILCs with traditional mesogens or mesogen-like moieties attached to the cation through a flexible spacer that decouples the two entities.

This study clearly demonstrates that the increased size of the core has a positive impact on the stabilization of the (SmA) mesophase over a wide temperature range. It therefore allowed for a complete study on how structural parameters influence the mesophase behavior. The large collection of synthesized ionic liquid crystals was compiled to investigate four key parameters: (i) the size and nature of aliphatic tails (including branching chains and ethylene glycol tails) **69**, **71**, **73**; (ii) the size of the mesogenic core **72**; (iii) lateral substitution on the mesogenic core **70**, **73**; and (iv) the nature of the counter ion **69**, **73**. The authors summarized that mesogens with larger cores display higher transitions temperatures, but the introduction of small lateral substituents on the imidazolium moiety can reduce the melting points. Too bulky lateral substituents, however suppress the mesomorphic behavior. Mesogens substituted with two long aliphatic tails showed common SmA phases and branching of the tails (either in a chiral or racemic form) has minimum effect on the mesophase behavior. Short tails use to promote SmA_2_ phases (interdigitated bilayers). Mesogens synthesized with ethylene glycol tails reduce the mesophase stability, due to the interaction of the lateral tail with the imidazolium cation. Doping the system with LiBF_4_ again stabilizes the mesomorphic phase and, additionally, is expected to improve anisotropic conductivity due to the presence of Li^+^ cations in the system. In this study, also the thermal behavior of different mixtures was explored. In some cases, the ionic mixtures show remarkable advantages compared to the pure compounds, for instance by suppressing crystallization and therefore reducing the viscosity of the system, both important to fulfil requirements for potential applications. In other mixture, however, the incompatibility of the SmA and SmA_2_ phases was observed.Parallel to the development of these rigid core imidazolium-based mesogens, also other approaches to combine ILCs with a more classical liquid crystal were followed. Central in this molecular design, again, is the connection of the imidazolium core to a mesogenic moiety, but now by a flexible linker. From a synthetic point of view, the introduction of a long flexible linker can be carried out by well-known routes; like simple esterifications or Williamson etherification reactions. Following this molecular strategy, compounds **74** and **75** (Figure 18) were synthesized. Compounds **74** with the nematogenic cholesteryl groups attached display a SmA* phases, but mesogens **75**, bearing cyanobiphenyl groups, displayed highly unexpectedly a (monotropic) nematic phase in the cooling ramp from isotropic liquid [14].

There is a limited amount of studies that relates the influence of the length of the flexible alkyl linker to the thermal behavior of the material. One example is based on imidazolium attached to a 2-phenylpyrimidine mesogen through different length spacers **76** [112]. With *para*-substitution and a C4 spacer, a SmA phase was found. Elongation of the spacer to C8 resulted in a small decrease of the melting temperature, but a significant decrease of the clearing temperature. SmC phases (also relatively rare for ILCs) were recorded for two of the variants with C8 spacer and longer side chains (*n* = 10, 12). The main motivation of this study was to develop room temperature ionic liquid crystals. One of the molecular design tools to accomplish this is based on a reduction of the symmetry that results in a decreased melting temperature. The authors selected a suitable approach for this purpose and used a geometry of the target calamitic 2-phenylpyrimidine via a *meta*-substitution, rather than the typical *para*-substitution [134,135]. The results showed that, indeed, this approach is successful and that *meta*-substitution at the phenyl ring resulted in room temperature mesophases with sufficient thermal stability. Replacing one of the rings by a carboxylate **77** leads to a significant decrease of the melting points, particularly for those containing a triflate counter ion.

**Figure 19:**
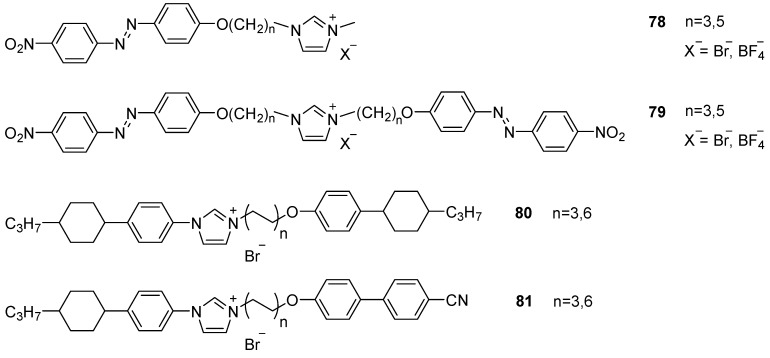
Imidazolium-based ILCs with light-sensitive azobenzene mesogen-like moieties (**78**,**79**) and hybrids with a rigid-core structure that is substituted at the second heteroatom position by a mesogenic group, connected through a medium or long spacer (**80**,**81**). Compound **81** exhibits the rarely observed enantiotropic nematic phase at, even at room temperature as would be an excellent lead for further exploration into applicable ILC materials.

Since the conformational freedom provided by these flexible chains showed unexpected results, a similar approach was targeted to link an azobenzene-based mesogen to the imidazolium core through a long alkyl chain (Figure 19, **78** and **79**) [136,137]. An additional attribute of materials containing azobenzene chromophores is that upon irradiation, the azobenzene double bond (reversibly) changes from a *trans* to a *cis* configuration. The reported mesomorphic behavior of these molecules shows melting points over 100 °C and narrow thermal ranges for the SmA mesophase. Their dicationic homologues, however, showed SmC phases. Recently, ILCs with both an extended mesogenic core as well as a mesogenic substituents **80** and **81** were published to show a rich polymorphism [138]. Mesogens with the cyanobiphenyl substituent **81** showed the rare enantiotropic nematic phase between ~40 and 100 °C. Furthermore, the authors mixed **81** (with *n* = 6) with chiral dopants to induce a cholesteric phase, a unique accomplishment.

### 4.3. Counter Ions

For a long time, the common approach in the field of ILCs was to study the anion independently from the mesogenic cation, although it was already discussed that a combined approach is more appropriate. The use of the well-stablished mesogen (cation!) tool box discussed in Section 4.1 and Section 4.2 (*i.e.*, manipulation of the core size, the number of tails, their nature and length, the presence of substituents, *etc*.) is not always sufficient to engineer desired properties in these mesomorphic materials [51]. Research in the field, also occasionally highlighted in the previous paragraph, shows the tremendous effect that the anion has on the physical properties of the material (viscosity, mesophases, transition temperatures, *etc*.) [40]. The counter ion should, therefore, be considered as an additional tool to efficiently modify the phase behavior [52,139,140].

The development of ILCs, followed that of the field of ionic liquids and primarily focused on counter ions with a strong hydrophobic nature (e.g., BF_4_^−^, PF_6_^−^, NTf_2_^−^, ClO_4_^−^, OTf^−^) besides the halides Cl^−^, Br^−^ and occasionally I^−^. Often general trends are found, such as the high transition temperatures for chloride salts and the very low ones for salts with the bis(triflamide) NTf_2_^−^ anions. The main limitation to uncover the role of the anion is that in these conventional series, the anions often vary in size, steric effects and charge distribution, resulting in a complex combined effect. Systematic work to dissect the possible steric and electronic contributions in different series of ILCs, so far is lacking. Therefore, one of the main priorities for maturing the field of ILCs is to expand the catalogue of possible anions and to define structure-property relations that define the contributions of these anions [141].

Additionally, the method chosen to introduce different anions needs to be reconsidered. Standard ion exchange procedures in the field are based on simple metathesis reactions. These procedures are highly suitable in the case of ionic liquid crystals bearing hydrophobic counter ions, which are stable enough to withstand the final purification steps. For hydrophilic anions like carboxylates, however, metathesis introduces purity challenges that are not readily solved by conventional synthetic procedures of ILCs [142,143,144]. Therefore, novel synthetic strategies that include mild purification steps (precipitation, re-crystallization, *etc*.) need to be explored. One such step, recently explored for ILs is based on the introduction of a hydrophilic anion during the formation of the cation [145]. Subsequent reactions to further modify the IL or ILC require conditions that are free from ionic contaminants, for instance halogens. In this work, the authors provide some examples of such transformations and also give routes to quantitative hydrophilic-hydrophilic ion exchange.

## 5. Conclusions and Outlook

The goal of this review was not to give a full listing of all prepared ionic liquid crystals, but rather to discuss the relationship between the molecular design and the mesomorphic properties of thermotropic ionic liquid crystals. Over time a wide range of organic cores has been used as the cation of an ILC, however we focused in this review on cationic cores that are either used a lot or that clearly demonstrate design principles. In the development of the mesogenic cations, considerable progress has been made and the community has defined a well-filled toolbox with different methods to manipulate the mesophase behavior. They include the size and nature (charge distribution) of the cation, its substitution pattern and the number and nature of the attached flexible tails. The counter ion has been underexposed and needs more attention in the years to come.

Ionic liquid crystals have been presented as mesomorphic materials with a rich polymorphism. In the majority of cases, this means a SmA phase, which can be bilayer or single layers either interdigitated or not. In literature, we occasionally find examples where different mesophases are reported: N, SmC, SmT, Col_h_ phases, *etc*. The field of ILCs would benefit from a broader knowledge of how to design these less common phases. Most particularly, the simple nematic phase may have the largest application potential, but is rarely observed in the ionic liquid crystal field and has just been registered for one type of ammonium-based mesogen. The search for more examples is likely to continue, in particular with functional and/or biocompatible counter ions.

A second design challenge in the field of ILCs is the formation of truly room temperature liquid crystals. This does not mean materials with high clearing temperatures that show no crystallization due to super-cooling. These materials that are commonly deep in the SmA phase display a highly solid-like character with associated high viscosities. ILCs with clearing points close to room temperature, or for biological applications close to 37 °C, have a much stronger fluid character in the application regime. Materials with such low clearing temperatures have been published for simple ammonium and imidazolium salts, but are much less prevalent in functional ILCs with for instance strong birefringence that require extended aromatic cores.

Finally, the field of ILCs would strongly benefit from defining a number of target applications. For conventional non-charged LCs, the discovery of display applications has accelerated the research on these materials, both from the application and from the fundamental side. Although suggestions for ILC applications are omnipresent, they have yet to prove themselves as truly functional materials. Examples of applications of ILCs are anisotropic tribology [146] and ion conductivity to be used in molecular electronics (solar cells, optoelectronic devices, *etc*.) [35,37,38,147,148], in directed synthesis approaches [149], including controlled nanoparticle synthesis, [150,151], alignment [152] or deposition [153], in ionic self-assembly [154,155] that builds complex structures from simple charged organic species and possibly in bio-catalysis [156] or sensing applications [25,98]. The near future will demonstrate which roles ILCs can play.

## Abbreviations

ArIm	arylimidazole or arylimidazolium
AmImAr	arylimidazolearyl or arylimidazoliumaryl
Col_h_, Col_r_	hexagonally, rectangular packed columnar (mesophase)
Cub_bis_	biscontinuous cubic liquid crystal
IL	ionic liquid
ILC	ionic liquid crystal
LC	liquid crystal
N	nematic (mesophase)
SmA, SmC, SmT	smectic A, smectic C or smectic T (mesophases)
SmA*	chiral smectic A (mesophase)
X^−^	anion
